# The smart senior care demand in China in the context of active ageing: a qualitative study with multiple perspectives

**DOI:** 10.3389/fpubh.2025.1505180

**Published:** 2025-04-04

**Authors:** Qiyuan Huang, Fang Liu, Song Ge, Shuang Teng, Xiang Wu, Xiao Zhang, Zhe Qu, Ying Li, Aming Wang, Mei Li, Xianping Tang

**Affiliations:** ^1^School of Nursing, Xuzhou Medical University, Xuzhou, China; ^2^Department of Natural Sciences/Nursing, University of Houston-Downtown, Houston, TX, United States; ^3^School of Medical Information Technology, Xuzhou Medical University, Xuzhou, China; ^4^Department of Pediatrics, Shandong Provincial Hospital Affiliated to Shandong First Medical University, Jinan, China; ^5^Affiliated Hospital of Xuzhou Medical University, Xuzhou, China; ^6^The People’s Hospital of Pizhou, Xuzhou, China; ^7^Aging Studies Institute of Xuzhou Medical University, Xuzhou, China

**Keywords:** smart senior care, active ageing, older adults, qualitative research, China

## Abstract

**Background:**

With the rapid population aging in China, smart senior care is urgently demanded. Therefore, it is necessary to comprehensively consider people’s demand for smart senior care for its better development. This study aimed to explore the needs and expectations of relevant stakeholders of smart senior care in China in the context of active aging.

**Methods:**

This is a qualitative descriptive study, in which 21 semi-structured interviews were conducted between October 2021 and March 2022. A total of seven community-dwelling older adults, seven smart senior care managers, and seven providers were selected using purposeful sampling. Interviews were transcribed, translated into English, and analyzed by the thematic analysis method.

**Results:**

Four major themes and 12 subcategories were identified in the data: “challenges of smart senior care” (low acceptance of users; high cost vs. low purchasing power; low coverage of smart devices and applications), “meet the demands of users” (strong medical care needs; meticulous life care needs; extensive social interaction needs), “multi-body participation” (online and offline integration; government’s broader role; cooperative gaming), and “all-around guarantee” (legal guarantee, technological empowerment, network security guarantee).

**Conclusion:**

To develop smart senior care in China in the context of active aging, it is essential to clarify the current issues and challenges faced by relevant stakeholders of smart senior care. To better develop smart senior care, we need to meet the health, life, and social care need of users, through multi-body participation and all-around guarantee.

## 1 Introduction

Population aging is accelerating in China. By the end of 2023, people aged 60 and older in China had reached 297 million, making up 21.1% of the world population. This number is expected to reach 34.1% by 2050 (1, 2). The aging process in China is far outpacing the most developing and developed countries; thus, older adults’ need for senior care in China is increasing. However, the low level of public services has caused a heavy senior care burden to families, communities as well as society, and traditional family care has been unable to meet the physical and mental health needs of older adults in China (3). Thus, there is an urgent need to transform and upgrade the traditional family care.

Against this backdrop, China has focused on combining Internet and senior care services. Smart senior care, which is defined as a senior care-based unit that deeply integrates Internet technology and home-based senior care services (4). This technology is available for sensing, predicting, reminding and responding to the older adults community’s daily requirements, assisting them in a timely and socially correct way (5, 6). With the development of technology and concepts, China has carried out four rounds of nationwide pilots on smart senior care (7). It has gradually been integrated into the family life of older adults, providing for independent and healthy senior care. In China, the diversification of senior care needs has led older adults to realize that they cannot rely solely on their children. The integration of information technology and traditional culture has not only enriched the lifestyles of older adults, but has also changed the social stereotypes of older adults and promoted their digital inclusion. In this cultural change, smart senior care has begun to take off in China. The existing research related to smart senior care mainly focuses on two aspects. One aspect is to focus on the development of intelligent technology and to assist older people to live independently by developing intelligent information systems and applying remote monitoring technology (8, 9). For example, Wang et al. (10) developed the Community-Based e-Health Program (CeHP) to help older adults manage their chronic conditions at home, promote quality of life, and reduce healthcare costs. The other aspect is to help analyze the demand for smart senior care services and clarify specific factors that affect users’ choice of smart senior care, helping provide more accurate service content (11, 12). For example, in a cross-sectional study, Huang et al. (11) identified the specific preference of older adults for smart senior care. Although the above research has certain value and significance, they seldom consider the needs of different stakeholders for smart senior care, including smart senior care managers and providers. In addition, we must acknowledge that current smart senior care in China still faces some challenges, such as low utilization rate due to the digital divide (13), structural mismatch, and low quality (5). Therefore, it is necessary to understand the viewpoints of multiple stakeholders on the future development of the smart senior care industry in China.

The Chinese government proposed a national strategy to actively address the aging population in 2019, encouraging the exploration of smart senior care development models from the active ageing perspective (14). The World Health Organization (WHO) defines “active ageing” as the fact that older adults can fully utilize their physical, mental, and social potential, and participate in society according to their needs, wishes, and abilities, to improve the quality of life. Meanwhile, older adults can obtain adequate protection and care whenever they need it. The concept contains three key elements-health, participation, and security (15). WHO encourages countries to integrate active ageing into practice when formulating senior care policies or implementing old-supporting services (15). Previous study had shown that, in the context of active ageing, the application of intelligent technology has a positive effect on the health, social participation, and life satisfaction of older adults (16). It can be seen that active ageing has potential advantages in guiding the development of smart senior care.

In China, research on the needs of smart senior care is still insufficient, and previous studies mainly focused on analysis from a single perspective, lacking comprehensive assessment from multiple perspectives (17–19). How to comprehensively and multi-dimensionally evaluate multiple stakeholders’ needs for smart senior care services remain to be explored. Therefore, this qualitative study aims to explore the demands and expectations of smart senior care from multiple stakeholders relevant to smart senior care in China using the perspective of active ageing, thereby providing a reference for the government, smart senior care managers and providers to take actions to improve the quality and effectiveness of smart senior care.

## 2 Methods

### 2.1 Design

A descriptive qualitative research method was used in this study. As this method can provide a low inference interpretation, which can help intuitively present and comprehensively summarize the experiences of various stakeholders in the context of smart senior care (20). During this study, we followed the standards for consolidated criteria for reporting qualitative studies (COREQ): a 32-item checklist (Additional file 1).

### 2.2 Participants and recruitment

Purposive sampling and snowball sampling were used to recruit participants from Jiangsu, China from October 2021 to March 2022. Based on the discussion of the research group, we finally identified three groups relevant to smart senior care, including smart senior care providers and managers as well as the older adults. We visited 2 civil affairs bureau on aging, 3 smart senior care companies, and 4 communities. To achieve maximum differential sampling, a maximum of 2 participants per unit were permitted. The recruitment of older adults was carried out with the assistance of smart senior care companies and communities staff. Additionally, smart senior care providers and managers were recruited by disseminating public information in We-Chat groups or email. Information shared included a letter of invitation, study purpose, eligibility criteria, potential risks, participation requirements, rights to confidentiality, consent, the right to withdraw from the study at any time, and the investigator’s contact information.

The criteria for participation included: (a) older adults are permanent residents of the community aged 60 and above, can express their ideas clearly, and have experience in smart senior care; (b) smart senior care managers, with intermediate or above professional titles, have experience in smart senior care management, and have been engaged in senior care management for 10 years or more; (c) smart senior care providers, with experience in smart senior care case services, and engaged in senior care services for 5 years or more. The sample size of participants was determined by data saturation where no new themes from participants’ experiences emerged (21). Table 1 presented the demographic characteristics of the participants.

**Table 1:** Participant characteristics (*N* = 21).

Variables	Older adults (*n* = 7)	Smart senior care manager (*n* = 7)	Smart senior care provider (*n* = 7)
Age (year)			
Mean (SD)	70.00 ± 4.96	42.71 ± 6.78	42.57 ± 5.80
Gender *N* (%)			
Male	4 (57.14%)	4 (57 14%)	3 (42.86%)
Education *N* (%)			
Senior high school or below	5 (71.43%)	-	2 (28.57%)
Bachelor degree	2 (28.57%)	4 (57.14%)	3 (42.86%)
Masters degree	-	3 (42.86%)	-
Doctorate	-	-	2 (28.57%)
Currently working setting *N* (%)			
Retirement	7 (100%)	-	-
Government	-	4 (57.14%)	-
Enterprise	-	3 (42.86%)	3 (42.86%)
Medical Institution	-	-	4 (57.14%)
Years of work experience *N* (%)			
0–5	-	-	-
6–10	-	-	2 (28.57%)
10–15	-	3 (42.86%)	2 (28.57%)
15–20	-	3 (42.86%)	1 (14.28%)
>20	-	1 (14.28%)	2 (28.57%)
Years of using smart senior care *N* (%)			
0–1	2 (28.57%)	-	-
1–2	1 (14.28%)	-	-
2–3	2 (28.57%)	-	-
>3	2 (28.57%)	-	-

### 2.3 Data collection

A semi-structured interview guide (Table 2) was designed and modified by five senior professionals with more than 10 years of experience in qualitative and gerontological research after group discussions. The interview questions were pretested with three participants (each of them was older person, care provider, and care manager for representation) to ensure the appropriateness and viability of the interview guide. The data of these three pre-interviews were not included in the final result (22). The purpose of the in-depth interview is to obtain the necessary information and have all participants provide comprehensive and detailed information about the phenomenon being studied.

**Table 2:** Interview guidelines.

Target population	Semi-structured interview guideline
Older adults	What are your needs or expectations for smart senior care?
	What smart senior care services do you want? Please explain in detail.
	What ways do you want to get smart senior care?
	What support conditions or guarantees do you want in the smart senior care?
Smart senior care manager	What do you think of the current development of smart senior care? What are the challenges?
	What stakeholders do you think are needed to participate and support smart senior care? Please explain their duties in detail.
	What do you think should be the focus on the development of smart senior care?
	What support conditions or guarantees do you think smart senior care needs?
Smart senior care provider	Please describe how your current job carries out smart senior care?
	What problems or challenges do you think exist in the smart senior care?
	What processes do you think need to be improved when providing smart senior care?
	What support conditions or guarantees do you think you need at smart senior care?

The in-depth interviews were conducted in a quiet room at a time convenient for the participants. Prior to the formal interview, the participants signed the informed consent which contained an explanation of the study’s objectives, procedure, and potential benefits, and risks. The interview sessions took about 40 to 65 min. The researchers began the interview with an informal talk. The purpose of this informal talk was to establish a trusting relationship with the participants. Meanwhile, we made sure that all interviewers had no previous relationship with the participants. All the interviews were conducted by the first author (QH), he is a master of nursing with background knowledge and practical experience in smart senior care, and proficient communication skills. During the interview, the first author tried his best to listen to the participants and observe at all times, adjust the interview rhythm, complete records, and maintain objectivity and neutrality. All interviews, including personal reflections and verbal and non-verbal behaviors observed during the interview, were transcribed verbatim by the first author and a research assistant (XT) within 24 h of the interviews and were reviewed by the first author for accuracy. The research assistant is a Doctor of Nursing with wealth of experience in the subject of geriatric nursing and qualitative research. Dependability was reflected in the debriefing sessions that were conducted after the pilot interviews.

### 2.4 Data analysis

The transcripts were imported into qualitative data analysis software ATLAS.ti 8. Each transcript was coded independently by QH and XT, and then each code application was discussed to reach consensus. Data analysis was performed using the thematic analysis method (23). The transcriptions were read several times, coded and condensed into meaning units and through interpretation of the content, main themes were identified. To increase the breadth and reliability of the analysis, a manual for coding was developed. Three authors (QH, FL and XT) discussed the themes in-depth until a consensus was reached on the relevant coded data in all themes. During the data analysis, all authors verified the results and the highlighted quotations. After analyzing the transcript of 21 interviewers, the research group identified that there was no further new information appeared, which indicated data saturation and data collection as well as data analysis was ended.

### 2.5 Study rigor

Based on the assessment criteria of Lincoln et al. (24), several methods were used to examine the credibility, transferability, dependability, and confirmability of data. For example, (a) investigator triangulation, (b) member checks, (c) peer debriefing, (d) searching for opposing evidence, and (e) holding regular meetings to discuss the data analysis process. The research methods were carefully developed so that the participants could follow the research process. All interviews were conducted in Chinese; then, the transcripts were translated into English. The transcripts were then reviewed by two bilingual researchers (SG and ZQ) independently.

### 2.6 Ethical approval

This study was approved by the Human-related Research Ethical Committee of the Affiliated Hospital of Xuzhou Medical University (XYFY2021-KL157-01). All methods were performed in accordance with the Declarations of Helsinki. The purpose, interview procedures, and timeline of the interview were explained to the participants before the study started. The participants were guaranteed anonymity and confidentiality and voluntary participation. All audio recordings were saved in a password-protected computer until each interview was transcribed and verified against the tapes.

## 3 Result

A total of 23 participants were recruited and 21 interviews were conducted with older adults (*n* = 7, four men), smart senior care managers (*n* = 7, four men), and smart senior care providers (*n* = 7, three men) which took place face-to-face. One older adult declined to participate in the study because of poor health, and one smart senior care provider drop out causing her high level of fatigue. Four theme categories emerged from the analysis of the interviews: challenges of smart senior care, meeting the demands of users, multi-body participation, and all-around guarantee.

### 3.1 Challenges of smart senior care in China

The participants stated that the unique characteristics of China’s smart senior care have brought challenges to its development. Poor social perception, high service price, and low coverage have influenced the popularity of smart senior care.

#### 3.1.1 The current lower acceptance of users

Users have difficulty understanding how smart senior care could contribute to their lives and have the challenge of a “digital divide” when using smart devices. The participants highlighted that guiding user to change their traditional care values is a key process to improve the popularization of smart senior care.

*“Although smart senior care has great benefits to users. However, Chinese older adults are limited in their ability to accept new care things and have different degrees of obstacles in the use of smart devices. These reasons lead to low acceptance and satisfaction of users to smart senior care. Therefore, we should make changes in the values of care, and guide older adults to accept smart senior care”* (Interview 12, care manager, government).

#### 3.1.2 High price vs. low purchasing power

During the study, the participants brought up an important factor hindering the popularization of smart senior care: the contradiction between expensive care services and the low consumption level of older adults. To address this issue, the participants suggested that the government should subsidize older care services. This would make smart senior care more affordable for a larger portion of the population.

*“At present, the price of smart senior care is too expensive, and I need to spend nearly 4,000 yuan (about 578 USD) on the care service every month. However, if the government were to integrate smart senior care into long-term care insurance, it would attract more older adults to buy this service”* (Interview 5, older adult, user).

#### 3.1.3 Low coverage of smart devices and applications

As smart senior care has just started in China, the current coverage of smart devices and applications in older adults is inadequate. Therefore, the participants expressed that it is imperative to enhance digital education amongst older adults and invest in infrastructure construction.

*“We are collaborating with communities to create a comprehensive smart senior care platform. Additionally, we are proactively educating senior residents on the use of smart devices and applications. Our goal is to provide more older adults with the peace of mind and convenience that comes with smart senior care”* (Interview 9, care manager, enterprise).

### 3.2 Meet the different demands of users

The demands of older adults for smart senior care have shown a diversified and personalized trend. Meeting the demands of different users and delivering accurate smart services is the goal of smart senior care. In this study, older adults’ demands for smart senior care can be summarized as (a) strong medical care needs, (b) meticulous life care needs, and (c) more social support needs.

#### 3.2.1 Strong medical care needs

Older adults are concerned about medical care services and having high-quality medical resources can promote the popularity of smart senior care among older adults. The participants expressed current difficulties in accessing medical care, as well as a strong desire for smart senior care.

*“It is inconvenient for older adults to go to the hospital. Especially in the face of sudden diseases, it is more difficult for them with mobility problems to seek medical attention in time. Therefore, I attach great importance to the utilization of mobile health technologies within the home setting, as they can provide invaluable assistance during critical moments”* (Interview 2, older adult, user).

#### 3.2.2 Meticulous life care needs

More meticulous life care will benefit more users. Smart senior care should be committed to meeting the fundamental and personalized needs of older adults for a healthy life through meticulous life care. The participants emphasized the need to broaden the content of life care.

*“Smart senior care focuses on more meticulous and personalized care services. Users have different life care needs, which requires care institutions to continuously enrich the content of life care. In addition to the provision of basic services such as nursing, cleaning and diet, the corresponding life care such as entertainment, travel, and social interaction should be added”* (Interview 16, care provider, enterprise).

#### 3.2.3 Extensive social interaction needs

Effective remote social interactions with family, friends, and caregivers are significant for older adults because they can share each other’s experiences at any time. The participants stated that extensive social interaction could facilitate their experience of using smart senior care.

*“I hope that it (smart senior care) can enrich my social interaction, so that I can maintain good interaction with my family and friends, with whom I would like to share life experiences. Of course, if I can communicate illness with my family physician all the time, that must be expected!”* (Interview 1, older adult, user).

### 3.3 Multi-body participation

Smart senior care is a collection of care resources consisting of multi-agency co-operative units. Active cooperation among social organizations can promote the maximization of both individual and societal interests. Multi-body participation is driven by three core contents (1) online and offline integration, (2) government’s broader role, and (3) cooperative game.

#### 3.3.1 Online and offline integration

The operation of smart senior care requires not only the information transmission of online systems but also the effective support of offline services. The participants pointed out that telemedicine alone is insufficient for assessing the health needs of older adults. Medical services such as diagnosis, treatment, and evaluation must be implemented offline to collaborate with the online system to provide high-quality services.

*“The supply of medical care services should be carried out simultaneously online and offline. We need to combine the doctor’s diagnostic information with on-site assessments to determine if their prescription is appropriate. Especially the operational diagnosis and treatment, which must be implemented offline”* (Interview 18, care provider, medical institution).

#### 3.3.2 Government’s broader role

The development of smart senior care in China is operated under a policy-driven, and therefore requires broader government involvement. Especially in coordinating the allocation of older care resources, standardizing the service process, and promoting product innovation, the government needs to play a more facilitating role. The participants emphasized that the government could establish more connections with all relevant stakeholders at different levels (such as information sharing, quality regulation, financial subsidies, etc.) to stabilize the consumer market and enable mutual benefits for all parties involved.

*“Government-led smart senior care can break the barriers of the “information fortress” in data interworking and resource sharing across multiple participants. … Meanwhile, the State Administration of Market Supervision needs to fulfill its market supervision responsibilities for smart senior care. Moreover, governments can increase the product innovation capacity of enterprises and the participation enthusiasm of consumers through financial incentives”* (Interview 10, care manager, government).

#### 3.3.3 Cooperative gaming

In Game Theory, cooperative gaming is concerned with stabilizing the operating mechanisms and benefits of multi-party synergies, and how to achieve optimal outcomes through cooperation. The participants proposed that social participation in smart senior care needs to be through a cooperative mechanism to connect stakeholders such as families, communities, governments, and enterprises. This will not only maximize the benefits for all the participants but also promote compatibility and establish a competitive and collaborative older care system.

*“Smart senior care involves many stakeholders. Limited resources can hardly guarantee that each participant can maximize their personal interests in it. Therefore, it is necessary to adopt a cooperative mechanism to create more public values together, to build an incentive-compatible, fair, and effective social support system”* (Interview 13, care manager, government).

### 3.4 All-round guarantee

Smart senior care is a dynamic evolutionary and continuous optimization process. The participants highlighted the realization of legal guarantee, technological empowerment, and network security as important link to ensure the sustainable development of smart senior care.

#### 3.4.1 Legal guarantee

The participants pointed out that legal guarantee is critical in smart senior care. The government regulates the smart senior care process and enhance the vitality of the market by formulating policies to form fair competition and regulated management among multiple participants eventually.

*“The improvement of the relevant legal system will promote the professional and standardized development of smart senior care. The government needs to establish industry standards around market access, financial support, and operation planning to create a market atmosphere of value co-creation”* (Interview 12, care manager, government).

#### 3.4.2 Technological empowerment

Artificial intelligence, the Internet of Things, and other modern technologies make the interaction between smart senior care and users more efficient and convenient. The participants expressed the importance of increasing technology empowerment and promoting research and development of key technology products.

*“Enterprises need to enrich the supply of smart products. In view of different application scenarios of smart senior care, health management wearable devices, portable health monitoring devices, and home service robots are developed to meet diversified and personalized needs for health care”* (Interview 20, care provider, enterprise).

#### 3.4.3 Network security guarantee

Network security has become a key concern of users when using smart senior care. The participants suggested that applying more advanced security technology and improving risk assessment are effective ways to protect information.

*“We are committed to protecting users’ privacy. In data collection and transmission, an advanced key system is used to encrypt and protect user data. Meanwhile, we constantly update the network intrusion detection technology to prevent system crash due to hacker attacks”* (Interview 8, care manager, enterprise).

## 4 Discussion

Overall, our results provide a deep understanding of the needs and visions of relevant stakeholders for the development of smart senior care under the concept of active ageing, and how to provide smart senior care throughout the process. The participants described how challenging it is to develop smart senior care in the condition of traditional Chinese culture and family. An important finding is that people hope that the government will include smart senior care in long-term care insurance, which will make care further affordable for more users. The needs of older adults for smart senior care tend to be diversified, prompting companies to focus on providing services such as health management, life care, and social interaction. Our results show that the government-led online and offline smart senior care model is more likely to promote multi-subject participation. At the same time, we innovatively found that the concept of cooperative games can be used to solve the problem of multi-subject benefit distribution. Finally, at the level of protection, legal protection, technical empowerment, and network security are the key content to ensure the sustainable development of smart senior care.

Our results illustrate the current challenges in developing smart senior care in China. First, the low acceptance of smart senior care among older adults affects its popularity, consistent with the research of Kong, D (17). Older adults’ gerontological changes have hampered their ability to accept new things. Research by Dermody et al. (25) demonstrated that according to users’ knowledge and experience level of smart technology, increasing the aging-appropriate design of products can make smart senior care more acceptable. Second, expensive service prices affect users’ willingness to purchase, which was also reported in a review (26). Smart senior care is a private customized service, and the high price makes it affordable for only a few users. Therefore, we suggest that the government can incorporate smart pensions into the long-term care insurance project to improve its universality. Users can enjoy high-quality smart senior care by paying the government-subsidized insurance amount regularly. Finally, the low coverage of smart devices and applications also limits the development of smart senior care. According to data reported by China Internet Network Information Center (CNNIC) in December 2021, about 43.2% of older adults in China use mobile phones to surf the Internet, and 80% of them actively seek Internet services (27). Therefore, we can moderately guide the teaching and use of smart senior care applications and increase the delivery of smart products among older adults.

The participants expressed the need for diversified smart senior care content, consistent with the results of an observational study (11). The study reported that older adults have a high demand for medical care, life care, and social entertainment services, and the physical condition and level of social support of the older adults are important factors affecting their willingness to purchase smart senior care. Previous studies have also pointed out that meeting the physical and mental health needs of older adults is an important opportunity for the development of smart senior care in China (4). Therefore, we suggest that enterprises should pay attention to the supply of health management, life care, social interaction, and other service content. At the same time, it is also necessary to assess the needs of users. Improvement of the care plan based on the unmet needs of users for the older adults can provide more accurate personalized intelligent senior care.

Multi-body participation is the premise of smart senior care to achieve win-win value. In this process, all participants can interdisciplinary integration, so that smart senior care resources can be effectively used and transformed. Firstly, the government should play a broader role in oversight and management. Previous research had emphasized the dominant position of the government in the process of policy formulation, resource allocation, and service management (26). Secondly, we should focus on flexibly applying smart senior care to the daily lives of older adults, improving their health through both online and offline channels. For example, Portugal has developed a virtual exercise system for older adult people living at home (28). This system not only guides older adults to exercise, but also monitors their health indicators and remotely reports the data to fitness coaches. Fu et al. (29) also pointed out that smart senior care combines online and offline services, which better activates the central position of value co-creation of older adults. Last but not least, we also discovered the potential advantages of applying cooperative gaming to smart senior care. A study in Italy also reported that the application of cooperative games in medical care can effectively solve the uneven distribution of resources and achieve the effect of saving financial budgets (30). In the smart senior care model, multiple subjects such as the government, community, senior care service providers and older adults can realize a win-win situation through the cooperative gaming mechanism. The government guides the participation of communities and enterprises in senior care services through the formulation of policies and the provision of incentives. The community as a hub connecting government and business is responsible for integrating demand and resources to drive the smart senior care platform to the ground. A series of cooperative gaming aims to enhance the vitality of the smart senior care market and achieve sustainable development.

The participants also indicated that legal protection, technological empowerment, and network security are related to the sustainable development of smart senior care. Previous studies had confirmed that a sound legal system can regulate the process of senior care services and control the quality of services (31). The Chinese government pointed out in the report on the development of smart senior care that enterprises should strengthen information technology support and improve product supply capabilities (32). Under the guidance of policies, enterprises should strengthen the innovation of technological products to adapt to different senior care scenarios. At the same time, Li et al. (33) emphasized the importance of privacy protection in the process of smart senior care. To address the issue of information security, a study by German scholars Zimmermann et al. (34) suggested that developers of smart senior care systems should design interfaces that are easy for users to understand and operate, as well as implement fallback mechanisms that enable users to take control of digital devices. Therefore, how to protect the legitimate rights and interests of consumers and improve the security of user information is also an important issue in the development of smart senior care in China.

### 4.1 Strengths and limitations

This study has several strengths. To start with, to the best of our knowledge, this is the first time to explore the needs and expectations of multi-stakeholder groups for smart senior care from the perspective of active ageing. In addition, we were able to conduct in-depth interviews, and thereby reach data saturation of the key concepts. Last but not least, we report according to the COREQ 32-item checklist, ensuring the rigor and credibility of our results.

However, we also acknowledge that the study has some limitations. On the one hand, although the relevant concepts have reached saturation, the sample size of each population group is relatively small, and the data collection is limited to the eastern part of China. On the other hand, we mainly get information from older adults who have rich experience in smart senior care. Thus, we do not know whether our findings apply to all older adults in China. In the future, more research on the demand for smart senior care with participants from wide sociodemographic regions in China is needed.

## 5 Conclusion

Our research analyzes the needs and expectations of relevant stakeholders for smart senior care from the perspective of active ageing. Participants described the multiple challenges of smart senior care in China and expressed potential needs for different types of services such as health, life, and social interaction. In addition, the participants provided a plan for the multi-subject participation form of smart pension. Moreover, various security measures for smart senior care are clarified. Our findings provide evidence for the development of smart senior care in China. In the future, it will be interesting to explore the construction of a smart senior care model in the context of active ageing.

## Data Availability

The raw data supporting the conclusions of this article will be made available by the authors, without undue reservation.
