# Supplementary material for: The smart senior care demand in China in the context of active ageing: a qualitative study with multiple perspectives
Source: Front Public Health. 2025 Apr 4;13:1505180. doi: 10.3389/fpubh.2025.1505180 (PMC12006124; doi:10.3389/fpubh.2025.1505180)
Supplement: Supplementary file 1 [file Data_Sheet_1.docx]

| **Consolidated criteria for reporting qualitative studies (COREQ): 32-item checklist** | | | |
| --- | --- | --- | --- |
| **No** | **Item** | **Guide questions/description** | **Reported on page No** |
| Domain 1: Research team and reflexivity | |  |  |
| 1 | Interviewer/facilitator | Which author/s conducted the interview or focus group? | P3 |
| 2 | Credentials | What were the researcher’s credentials? | P3 |
| 3 | Occupation | What was their occupation at the time of the study? | P3 |
| 4 | Gender | Was the researcher male or female? | P3 |
| 5 | Experience and training | What experience or training did the researcher have? | P3 |
| 6 | Relationship established | Was a relationship established prior to study commencement? | P3 |
| 7 | Participant knowledge of the interviewer | What did the participants know about the researcher? | P3 |
| 8 | Interviewer characteristics | What characteristics were reported about the interviewer/ facilitator? | P3 |
| Domain 2: study design | |  |  |
| 9 | Methodological orientation and Theory | What methodological orientation was stated to underpin the study? | P2 |
| 10 | Sampling | How were participants selected? | P2 |
| 11 | Method of approach | How were participants approached? | P2 |
| 12 | Sample size | How many participants were in the study? | P4 |
| 13 | Non-participation | How many people refused to participate or dropped out?Reasons? | P4 |
| 14 | Setting of data collection | Where was the data collected? | P3 |
| 15 | Presence of non-participants | Was anyone else present besides the participants and researchers? | P3 |
| 16 | Description of sample | What are the important characteristics of the sample? | Table 1 |
| 17 | Interview guide | Were questions, prompts, guides provided by the authors? Was it pilot tested? | P3 |
| 18 | Repeat interviews | Were repeat interviews carried out? If yes, how many? | Not applicable |
| 19 | Audio/visual recording | Did the research use audio or visual recording to collect the data? | P4 |
| 20 | Field notes | Were field notes made during and/or after the interview or focus group? | P4 |
| 21 | Duration | What was the duration of the interviews or focus group? | P3 |
| 22 | Data saturation | Was data saturation discussed? | P4 |
| 23 | Transcripts returned | Were transcripts returned to participants for comment and/or correction? | P4 |
| Domain 3: analysis and findings | |  |  |
| 24 | Number of data coders | How many data coders coded the data? | Not applicable |
| 25 | Description of the coding tree | Did authors provide a description of the coding tree? | Not applicable |
| 26 | Derivation of themes | Were themes identified in advance or derived from the data? | P4 |
| 27 | Software | What software, if applicable, was used to manage the data? | P4 |
| 28 | Participant checking | Did participants provide feedback on the findings? | P4 |
| 29 | Quotations presented | Were participant quotations presented to illustrate the themes / findings? Was each quotation identified? | P5, Result |
| 30 | Data and findings consistent | Was there consistency between the data presented and the findings? | P5, Result |
| 31 | Clarity of major themes | Were major themes clearly presented in the findings? | P5, Result |
| 32 | Clarity of minor themes | Is there a description of diverse cases or discussion of minor themes? | P5, Result |

Note: P: page
